# The relationship between homoarginine and liver biomarkers: a combination of epidemiological and clinical studies

**DOI:** 10.1038/s41598-023-32363-4

**Published:** 2023-03-30

**Authors:** Ali Aghdassi, Edzard Schwedhelm, Dorothee Atzler, Matthias Nauck, Jens-Peter Kühn, Marie-Luise Kromrey, Henry Völzke, Stephan B. Felix, Marcus Dörr, Till Ittermann, Martin Bahls

**Affiliations:** 1 Department of Medicine A – Gastroenterology, Nephrology, Endocrinology and Rheumatology, University Medicine Greifswald, Greifswald, Germany; 2 Institute of Clinical Pharmacology and Toxicology, University Medical Center Hamburg-Eppendorf, Hamburg, Germany; 3 DZHK (German Center for Cardiovascular Research), Partner Site Hamburg, Hamburg, Germany; 4 Institute for Cardiovascular Prevention, Ludwig-Maximilians-Universität, Munich, Germany; 5 DZHK (German Centre for Cardiovascular Research), Partner Site Munich Heart Alliance, Munich, Germany; 6 Walther Straub Institute of Pharmacology and Toxicology, Ludwig-Maximilians-Universität, Munich, Germany; 7 Institute of Clinical Chemistry and Laboratory Medicine, University Medicine Greifswald, Greifswald, Germany; 8 Institute and Policlinic for Diagnostic and Interventional Radiology, University Hospital, Carl Gustav Carus University, TU Dresden, Dresden, Germany; 9 Institute for Radiology and Neuroradiology, University Medicine Greifswald, Greifswald, Germany; 10 DZHK (German Center for Cardiovascular Research), Partner Site Greifswald, Greifswald, Germany; 11 Institute for Community Medicine, University Medicine Greifswald, Greifswald, Germany; 12 Department of Internal Medicine B, University Medicine Greifswald, Sauerbruchstr, 17475 Greifswald, Germany

**Keywords:** Diagnostic markers, Prognostic markers

## Abstract

Homoarginine (hArg) is a non-essential cationic amino acid which inhibits hepatic alkaline phosphatases to exert inhibitory effects on bile secretion by targeting intrahepatic biliary epithelium. We analyzed (1) the relationship between hArg and liver biomarkers in two large population-based studies and (2) the impact of hArg supplementation on liver biomarkers. We assessed the relationship between alanine transaminase (ALT), aspartate aminotransferase (AST), γ-glutamyltransferase (GGT), alkaline phosphatases (AP), albumin, total bilirubin, cholinesterase, Quick’s value, liver fat, and Model for End-stage Liver Disease (MELD) and hArg in appropriately adjusted linear regression models. We analyzed the effect of L-hArg supplemention (125 mg L-hArg daily for 4 weeks) on these liver biomarkers. We included 7638 individuals (men: 3705; premenopausal women: 1866, postmenopausal women: 2067). We found positive associations for hArg and ALT (β 0.38 µkatal/L 95% confidence interval (CI): 0.29; 0.48), AST (β 0.29 µkatal/L 95% CI 0.17; 0.41), GGT (β 0.033 µkatal/L 95% CI 0.014; 0.053), Fib-4 score (β 0.08 95% CI 0.03; 0.13), liver fat content (β 0.016% 95% CI 0.006; 0.026), albumin (β 0.030 g/L 95% CI 0.019; 0.040), and cholinesterase (β 0.003 µkatal/L 95% CI 0.002; 0.004) in males. In premenopausal women hArg was positively related with liver fat content (β 0.047% 95%CI 0.013; 0.080) and inversely with albumin (β − 0.057 g/L 95% CI − 0.073; − 0.041). In postmenopausal women hARG was positively associated with AST (β 0.26 µkatal/L 95% CI 0.11; 0.42). hArg supplementation did not affect liver biomarkers. We summarize that hArg may be a marker of liver dysfunction and should be explored further.

## Introduction

Homoarginine (hArg), a homologue of L-arginine, is a non-essential cationic amino acid, and is synthesized from arginine and lysine by arginine:glycine amidinotransferase (AGAT) and possibly enzymes of the urea cycle^1,2^. This amino acid is synthesized mainly in the liver, but also in the kidneys, brain, and small intestine, organs which also express AGAT^1,3^. AGAT is involved in energy metabolism by catalyzing the first and rate-limiting step in creatine synthesis^4^. Being a weak substrate for nitric oxide (NO) synthase hArg competes with L-arginine for NO synthesis^5^ thereby contributing to vascular homeostasis^6^. Experimental^7^ and clinical studies^8,9^ have shown that low endogenous hArg levels in the circulation are associated with cardio- and cerebrovascular events and all-cause mortality^10^.

Of particular note, hArg is a non-competitive inhibitor of alkaline phosphatases (APs), a group of enzymes that hydrolyze orthophosphoric-monoester in alkaline pH and consist of different subtypes expressed in several mammalian tissues, among them liver, bone, kidney, intestine, and placenta^11,12^. The human bone and liver APs are most strongly inhibited by hArg^11^. AP exerts inhibitory effects on bile secretion in the liver by targeting intrahepatic biliary epithelium thus having a protective effect on the liver tissue as a further increase of bile pressure is avoided^13^. The relationship between liver function and hArg release is currently unclear. Low serum hArg concentrations predicted higher long-term mortality in patients with liver cirrhosis and correlated with model of end-stage liver disease (MELD), a score that assesses urgency of liver transplantation^14^. The relevance of arginine metabolism on liver function is further supported by the observation, that other metabolites such as asymmetric dimethylarginine (ADMA) and symmetric dimethylarginine (SDMA) are related to acute and chronic liver disorders^15–17^.

Here we analyzed (1) the relationship between liver biomarkers and hArg in the large population based epidemiological Study of Health In Pomerania (SHIP) and (2) the impact of hArg supplementation on liver biomarkers in healthy individuals.

## Material and methods

### Study population

For the analyses, we used data from two independent cohorts of the Study of Health In Pomerania (SHIP)^18^ and from a cross-over trial with L-hArg supplementation^19^. The SHIP project includes several large population-based studies, which were all conducted in the Northeast of Germany. In the first SHIP cohort; SHIP-START, 6265 individuals aged 20–79 years were selected from population registries, of which 4308 individuals (response 68.8%) participated between 1997 and 2001^20^. Between 2008 and 2012 baseline examinations of a second cohort (SHIP-TREND) were conducted. For SHIP-TREND a separate stratified random sample of 8826 adults aged 20–79 years was drawn and 4420 subjects participated (response 50.1%). For the present analyses, we used data from the baseline examinations of SHIP-START-0 and SHIP-TREND-0. In both studies all participants gave written informed consent. The studies were approved by the Local Ethics Committee of the University of Greifswald and comply with Declaration of Helsinki. Of the 8728 individuals, who participated in SHIP-START-0 or SHIP-TREND-0, we excluded 1008 individuals with missing data in any of the considered variables and 74 individuals with hArg concentrations higher than 6 µmol/L resulting in a final study population of 7646 individuals.

The cross-over trial was conducted in healthy individuals, which were recruited from the staff of the University Medical Centre Hamburg-Eppendorf, as described previously^19^. In brief, after inclusion (baseline), participants were randomized to supplement or placebo, i.e. 125 mg L-hArg or lactose once daily for 4 weeks, respectively. After a wash-out phase of 28 days, participants were switched to the other treatment. Finally, participants were examined 28 days after the discontinuation of the second treatment (follow-up). Alanine transaminase (ALT), aspartate aminotransferase (AST), and AP were determined with routine laboratory assays. The study was conducted as a non-drug study and the study protocol has been approved by the Ethics Committee of the Hamburg board of Physicians (PV4038) accordingly. The investigation was conducted in accordance with the Declaration of Helsinki and registered at clinicaltrials.gov (NCT02675660).

### Assessments in SHIP

Socio-demographic characteristics and menopause status were assessed by computer-assisted personal interviews. Mean daily beverage-specific alcohol (beer, wine, and distilled spirits) consumption was determined from alcohol intake during the last thirty days preceding the examination. Subjects who participated in exercise training during summer or winter for at least 1 h per week were classified as being physically active. Height and weight were measured to calculate the body mass index (BMI = weight (kg)/height^2^ (m^2^)). Waist circumference was measured to the nearest 0.1 cm using an inelastic tape midway between the lower rib margin and the iliac crest in the horizontal plane with the subject standing comfortably with weight evenly distributed on both feet.

Blood samples were taken non-fasting in SHIP-START-0. In SHIP-Trend 75% of the blood samples were taken in the fasted state. All samples were collected between 7 a.m. and 2 p.m. and analysed in the Institute of Clinical Chemistry and Laboratory Medicine of the University Medicine Greifswald. Alanine transaminase (ALT), aspartate aminotransferase (AST), γ-glutamyltransferase (GGT), LDL-cholesterol (LDL-C), HDL-cholesterol (HDL-C), triglycerides, creatinine, AP, albumin, total bilirubin, and cholinesterase were measured in serum on the Dimension Vista 1500 analytical system (Siemens Healthcare GmbH, Eschborn, Germany). The fibrosis (FIB-4) score was calculated using the formula: age*AST/(platelets*sqrt(ALT))^21^. Model for end-stage liver disease (MELD) was calculated using the following formula: 3.8*log_e_(serum bilirubin [mg/dL]) + 11.2*log_e_(INR) + 9.6*log_e_(serum creatinine [mg/dL]) + 6.4. High and low biomarker levels were defined according to the reference limits provided by our laboratory (ALT: > 0.77 µkatal/L in males and > 0.60 µkatal/L in females; AST: > 0.59 µkatal/L; GGT: > 0.96 µkatal/L in males and > 0.65 µkatal/L in females; AP: > 2.26 µkatal/L; albumin: < 34 g/L; bilirubin: > 17 µmol/L; cholinesterase: > 316 µkatal/L in males and women > 40 years; > 262 µkatal/L in women ≤ 40 years).

HArg was determined at the University Medical Centre Hamburg-Eppendorf by LC–MS/MS^22^. In brief, 25 µL of serum were diluted with stable isotope labeled internal standard ([^13^C_6_]-homoarginine). Subsequently, proteins were precipitated with methanol and guanidine compounds were converted to their butyl esters. Concentrations were calculated with calibration curves (four levels, triplicates), and platewise quality controls were run (two levels, duplicates). Intra- and interassay coefficients of variation was ≤ 7.5%.

Transabdominal ultrasound of the liver was performed by examiners using a B-mode ultrasound device (vivid I; GE-Healthcare, Waukesha, WI, USA) with a 2.5 MHz ultrasonic transducer. The examiners used a 2-point scale to assess the presence of hepatic steatosis: (0) no steatosis and (1) steatosis. Hepatic steatosis was defined as a hyperechogenic liver pattern.

In SHIP-TREND-0 liver MRI was performed without intravenous contrast using a 1.5-Tesla MRI system (Magnetom Avanto, VB15; Siemens Healthineers, Erlangen, Germany) with a 12-channel-phased-array surface coil^23^. Three-dimensional chemical shift encoded gradient-echo data with three echoes and flyback readout gradient were acquired from an axial slab during a single 19-s breath hold. Offline reconstructions of a PDFF map (including correction for T1 bias and T2* decay) and a R2* map (based on T2* decay measurement of PDFF) were performed^24^. Parametric maps of PDFF and R2* were used for further analyses. Mean PDFF was determined at operator-defined regions of interest placed at the center of the liver, by using Osirix (v3.8.1; Pixmec Sarl, Bernex, Switzerland)^23^.

### Statistical methods

Stratified by AST levels continuous data were reported as median, 25th, and 75th percentile, while categorical data were reported as percentage. All multivariable analyses were conducted stratified by sex or menopausal status for the female study participants. Associations of liver biomarkers and AP as exposure variables with hArg levels as outcome were analysed by linear regression models for each exposure separately adjusted for age, waist circumference, alcohol consumption, physical activity, triglycerides, HDL-C, serum creatinine, and study. Assumptions of the linear regression model were visually inspected by QQ and residual plots. In none of the models the assumptions for the linear regression model were violated. To compare the effect sizes of the different biomarkers on hArg we repeated the regression analyses using percentile values of the biomarkers.

In the cross-over trial we plotted the individual time courses of ALT and AST concentrations during the study at the visits baseline, after L-hARG supplementation, after placebo supplementation, and at follow-up. Differences in AST and ALT concentrations between different time points were compared by signed rank tests. All analyses were conducted with Stata 17.0 (Stata Corporation, College Station, TX, USA).

## Results

There were 396 individuals (5.2%) with high AST levels (Table 1). Individuals with high AST were older, more often males, drank more alcohol and had a higher BMI than individuals with normal AST. hArg levels were in median 0.21 µmol/L higher in the group with high AST. The median of AST, ALT, GGT, AP, fibrosis score, and liver fat content were in median higher in the high AST group.

**Table 1:** Characteristic of the study population stratified by aspartate amino-transferase (AST) concentrations.

	N	AST ≤ 0.59 µkatal/L (n = 7250)	AST > 0.59 µkatal/L (n = 396)
Age; years	7646	51 (37; 64)	54 (42; 63)
Sex			
Men	7646	47.1%	74.5%
Premenopausal women		25.5%	5.8%
Postmenopausal women		27.5%	19.7%
Physically active	7646	57.9%	53.3%
Alcohol consumption; g/day	7646	4.0 (0.7; 11.7)	11.3 (1.7; 32.4)
Body mass index; kg/m^2^	7646	27.0 (24.0; 30.4)	29.2 (25.9; 33.0)
Waist circumference; cm	7646	89 (79; 99)	98 (89; 108)
LDL-cholesterol; mmol/L	7676	3.3 (2.7; 3.9)	3.4 (2.6; 4.0)
HDL-cholesterol; mmol/L	7646	1.33 (1.10; 1.61)	1.23 (0.99; 1.57)
Triglycerides; mmol/L	7646	1.40 (0.97; 2.05)	1.90 (1.20; 2.99)
Creatinine; µmol/L	7646	80 (70; 90)	84 (74; 93)
Homoarginine; µmol/L	7646	2.61 (2.07; 3.30)	2.83 (2.23; 3.49)
Alanine amino transferase; µkatal/L	7646	0.36 (0.28; 0.48)	0.88 (0.64; 1.20)
High ALT		6.6%	65.7%
Aspartate-amino transferase; µkatal/L	7646	0.30 (0.24; 0.36)	0.73 (0.65; 0.94)
Gamma-glutamyl transferase; µkatal/L	7629	0.49 (0.38; 0.70)	1.20 (0.75; 2.70)
High GGT		17.8%	69.2%
Alcalic phospatase; µkatal/L	7517	1.10 (0.93; 1.40)	1.30 (1.10; 1.60)
High AP		0.8%	5.1%
Fibrosis score	7643	0.93 (0.63; 1.31)	1.72 (1.26; 2.32)
Hyperechogenic pattern in ultrasound	7646	32.8%	68.4%
Liver fat content; %	1849	3.9 (2.3; 7.8)	10.1 (3.7; 20.9)
> 5.1%		38.7%	67.1%
Albumin; g/L	7533	40 (38; 42)	41 (39; 43)
Low albumin		1.3%	3.1%
Total bilirubin; µmol/L	6613	7.0 (5.3; 9.2)	8.5 (6.3; 11.3)
High bilirubin		3.4%	8.0%
Cholinesterase; µkatal/L	7525	206 (178; 234)	222 (188; 253)
High cholinesterase		0.6%	2.0%
Quick’s value; %	7206	106 (98; 114)	104 (95; 114)
Model of end stage liver disease (MELD) score	6199	6.21 (6.21; 6.93)	6.37 (6.21; 7.18)

Data are expressed medians, 25th, and 75th percentiles (continuous data) or as percentages (categorical data).

**Figure 1:**
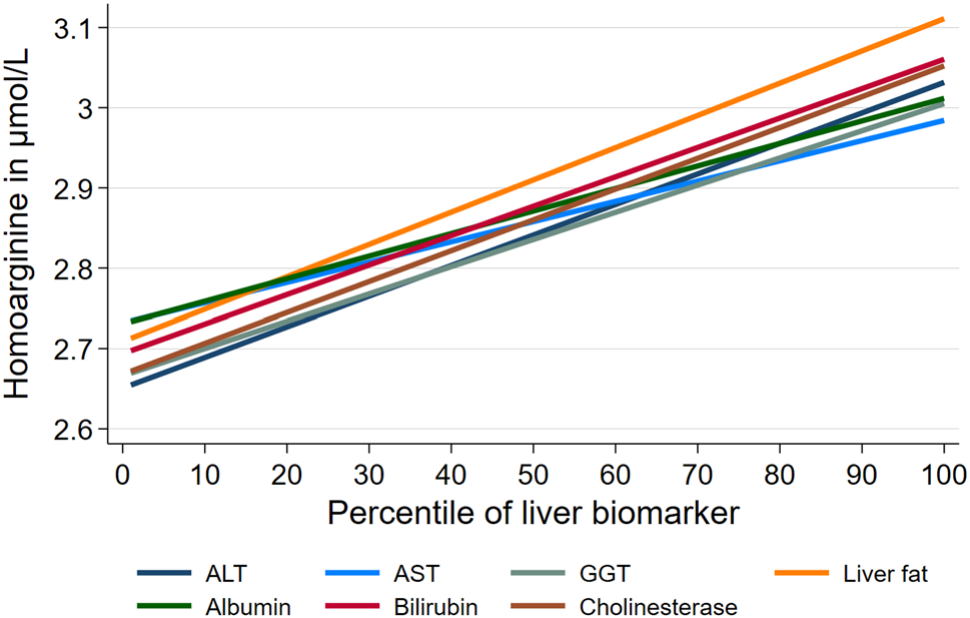
Association of liver biomarkers (expressed in percentiles) with hArg adjusted for age, waist circumference, alcohol consumption, physical activity, triglycerides, HDL-cholesterol, serum creatinine, and study in males.

In the adjusted multivariable analyses, we found positive associations of ALT, AST, GGT, Fib-4 score, liver fat content, albumin, bilirubin, and cholinesterase with hArg concentrations in males (Table 2). Associations were strongest for AST and cholinesterase concentrations (Fig. 1). In premenopausal women we observed positive associations of high GGT and liver fat content with hArg concentrations, whereas albumin was inversely associated with hArg. In postmenopausal women only ALT was positively associated with hArg concentrations.

**Table 2:** Association of liver biomarkers with homoarginine.

	Men (n = 3705)	Premenopausal women (n = 1866)	Postmenopausal women (n = 2067)
	β (95% confidence interval)		
Alanine amino transferase (ALT); µkatal/L	0.38 (0.29; 0.48)*	0.26 (− 0.06; 0.58)	0.26 (0.11; 0.42)*
High ALT	0.24 (0.16; 0.33)*	0.10 (− 0.14; 0.34)	0.10 (− 0.02; 0.23)
Aspartate-amino transferase (AST); µkatal/L	0.29 (0.17; 0.41)*	0.32 (− 0.12; 0.76)	0.17 (− 0.05; 0.39)
AST > 0.59 µkatal/L	0.13 (0.02; 0.23)*	− 0.18 (− 0.59; 0.24)	0.03 (− 0.15; 0.21)
Gamma-glutamyl transferase (GGT); µkatal/L	0.033 (0.014; 0.053)*	0.143 (− 0.049; 0.336)	− 0.005 (− 0.063; 0.052)
High GGT	0.14 (0.07; 0.21)*	0.16 (0.01; 0.32)*	0.01 (− 0.08; 0.09)
Fibrosis score	0.08 (0.03; 0.13)*	− 0.06 (− 0.30; 0.18)	0.07 (− 0.02; 0.15)
Liver fat content; %	0.016 (0.006; 0.026)*	0.047 (0.013; 0.080)*	0.007 (− 0.004; 0.019)
Liver fat content > 5.1%	0.23 (0.09; 0.36)*	0.26 (− 0.06; 0.59)	0.14 (− 0.02; 0.31)
Hyperechogenic pattern in ultrasound	0.16 (0.10; 0.23)*	0.07 (− 0.07; 0.22)	0.05 (− 0.03; 0.13)
Albumin; g/L	0.030 (0.019; 0.040)*	− 0.057 (− 0.073; − 0.041)*	− 0.002 (− 0.014; 0.011)
Total bilirubin; µmol/L	0.016 (0.009; 0.023)*	0.005 (− 0.008; 0.019)	0.005 (− 0.007; 0.016)
Total bilirubin > 17 µmol/L	0.10 (− 0.04; 0.24)	0.07 (− 0.24; 0.39)	− 0.04 (− 0.32; 0.23)
Cholinesterase; µkatal/L	0.003 (0.002; 0.004)*	− 0.000 (− 0.002; 0.001)	0.001 (− 0.000; 0.002)
Quick’s value; %	0.001 (− 0.001; 0.003)	0.004 (0.001; 0.009)*	− 0.001 (− 0.003; 0.002)
Model of end stage liver disease (MELD) score	− 0.019 (− 0.034; − 0.005)*	− 0.031 (− 0.117; 0.055)	− 0.002 (− 0.025; − 0.021)

Results are derived from linear regression models with serum homoarginine levels as outcome adjusted for age, waist circumference, alcohol consumption, physical activity, triglycerides, HDL-cholesterol, serum creatinine, and study. Analyses were conducted for each exposure separately.

*p < 0.05.

In men and postmenopausal women, we identified inverse associations between hArg and AP concentrations (men: β = − 0.020, 95% confidence interval [CI] − 0.032 to − 0.007, p = 0.002; postmenopausal women: β = − 0.035, 95% CI − 0.056 to − 0.015, p = 0.001). In premenopausal women we could not show such an association (β = 0.003, 95% CI − 0.010 to 0.017, p = 0.614). Quick’s value was positively associated with hArg in premenopausal women only (β = 0.004, 95% CI 0.001 to 0.009, p = 0.042). MELD was inversely related with hArg in men only (β = − 0.019, 95% CI − 0.034 to − 0.005, p = 0.002).

**Figure 2:**
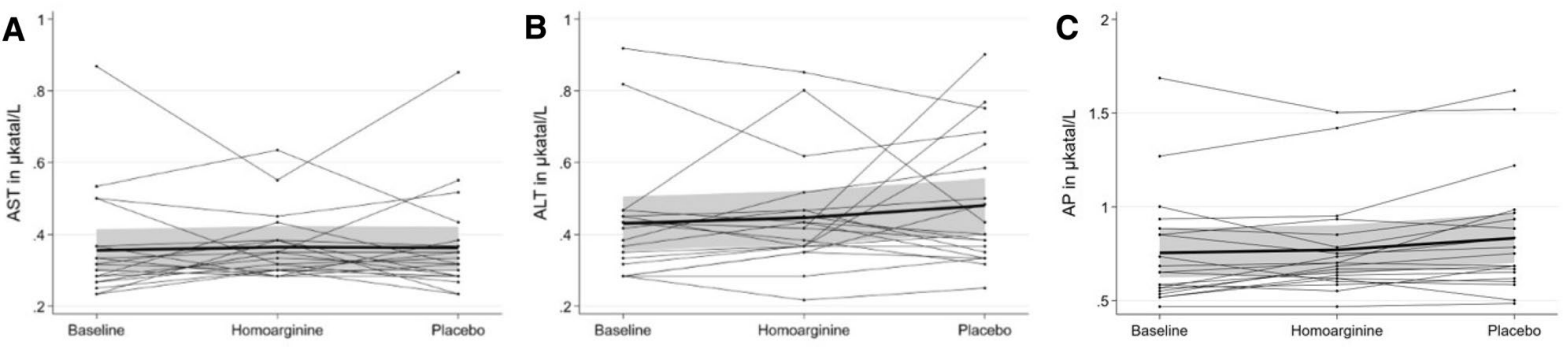
Individual time course of ALT (**A**), AST (**B**) and AP (**C**) levels over the difference phases of the cross-over trial. The thick lines represent the mean time course.

In the cross-over trial we found no increase of mean ALT or AST concentrations after supplementation with L-hArg (Fig. 2). Compared to baseline ALT and AST concentrations were not significantly higher at the end of the L-hArg supplementation phase (p = 0.588 for ALT and p = 0.269 for AST). Likewise, values differed not significantly between end of the L-hArg supplementation phase and end of the placebo phase (p = 0.685 for ALT and p = 0.618 for AST). AP was not influenced by hArg supplementation (Fig. 2).

## Discussion

Here we report that abnormal liver biomarkers are related to greater circulating hArg concentrations in the general population. We further report that hArg supplementation did not alter liver markers in healthy individuals. We believe that these two results are not contradictive but rather imply a potentially causal relationship between hArg measured in the circulation and liver function. Classical studies from the 1970s already highlighted the important role for hArg as a potent inhibitor of human hepatic AP^11,25^. While we acknowledge that much greater concentrations of hArg are required to inhibit AP in hepatocytes, we could not measure hArg in hepatocytes in an epidemiological setting. We also report that greater hArg concentrations were related to lower liver AP in men and post-menopausal women. While one may consider our finding that lower AP concentrations are related to higher hArg as potentially counterintuitive, it is important to distinguish between hepatocellular disease and cholestatic patterns, although the latter might also co-occur in advanced liver disorders. Hepatocellular disease is characterized by greater ALT and AST out of proportion of AP and bilirubin. Cholestatic patterns are characterized by higher AP and bilirubin compared to ALT and AST^26^. Taken together, the greater hArg concentrations may inhibit hepatic AP. Hence, hArg may be a marker for subclinical liver damage. Since hArg supplementation which increased circulating hArg levels did not induce liver abnormalities, we speculate that greater hepatic hArg synthesis by the liver may be a compensatory mechanism for liver damage.

Assessment of hepatic function in clinical routine is done by several ways but each of them bears their own limitations. They consist of measurements of single liver parameters including ALT or AST, markers of cholestasis, albumin, and prothrombin time or combinations of blood parameters in form of various indices such as the FIB-4 score^27^. Imaging-based assessment of the liver, i.e. by ultrasound or MRI can provide additional information on liver pathologies and functional volume. The patterns of abnormalities in liver function tests enable the differentiation into hepatocellular defects or biliary tract pathologies or combinations of them. Elevations of ALT and AST, two enzymes responsible for amino acid catabolism and ATP-production, indicate hepatocellular damage, while AST is less specific due to a considerable expression in other organs besides the liver^28^. Moreover, a disproportionate increase of ALT and AST is often observed, which results from their different intracellular concentrations. In contrast to ALT, exclusively localized in the cytoplasm, AST is also found inside mitochondria. A rise in AST indicates a more severe damage to hepatocytes such as alcohol-induced toxicities^29^. On the other hand, elevations in bilirubin and GGT are indicative of cholestatic diseases although the latter one may also be enhanced following cytochrome-inducing drugs or alcohol abuse^28^.

Our results show a positive correlation of hArg with both transaminases, GGT, and bilirubin in males while no significant associations were observed in females. In addition, hArg was related with liver fat content estimated by MRI in both men and women but this association only remained significant in males in conditions with higher liver fat content exceeding 5.1%. These findings lead to the assumption that in men higher endogenous serum hArg concentrations seem to correlate with both parenchymal, i.e. hepatocellular, or biliary damage and contradict investigations done in patients with advanced liver diseases where low hArg levels indicated impaired liver function^14^. However, our study population did not include individuals with progressed liver disorder and liver enzyme variations might rather indicate subclinical changes of liver function. One may hypothesize that hArg elevations result from a counter-regulative effect leading to an induction of synthesis of the vasodilator NO. This gas subsequently improves perfusion of intestinal organs, such as the liver. Hepatoprotective effects and improvements of vascular perfusion by NO have been demonstrated in experimental studies^30,31^. In addition, the identified diverse correlations between sexes with regards to liver markers and hArg deserves further research. However, albumin is a known binding partner of female sex steroid hormones and its synthesis is altered by estradiol^32^ indicating that indirect effects of estrogens and other female sex hormones might co-exist. In this respect additional investigations are necessary.

Oral supplementation of healthy humans with 120 mg L-hArg once daily for 4 weeks increased the plasma concentration several fold in the cross-over trial^19^. In contrast to the positive association of hArg with liver enzymes in the SHIP cohort, we did not observe an increase of liver enzymes after oral supplementation in the cross-over trial. This observation further substantiates the hypothesis that hArg elevations are rather a consequence than a cause of liver enzyme elevations in SHIP. hArg is a constituent of diet including some pulses^33,34^. We believe that participants of SHIP unlikely have adapted their dietary habits to a more hArg-rich food depending on liver enzyme status. Unfortunately, we do not have sufficient dietary records from our SHIP participants. hArg is extensively metabolized in the liver, kidney and other organs^1^. Catabolism of hArg is catalyzed by AGAT and degradation of hArg is catalyzed by AGXT2^35^. Moreover, hArg is a substrate and competitive inhibitor of arginases^1^. In particular, the expression of these enzymes in the liver might be regulated in dependence of the liver function. Therefore, it seems likely that either the synthesis of hArg in the liver is elevated or the degradation of hArg in the liver is impaired with increasing circulating concentrations of liver enzymes.

The FIB-4 score integrates the patient’s age, AST, ALT, and platelets and was initially developed for prediction of fibrosis in HCV/HIV co-infected patients^36^. However, unintentionally this score was useful for fibrotic liver diseases of other origin as well^37^. Albeit, liver biopsies remain the gold standard for assessment of severity of liver disease. We found a positive correlation of hArg with FIB-4 only for male study participants. The non-significant association between the majority of standard liver functions tests and hArg remains elusive and data are sparse. Sex-dependent differences of metabolites, among them hArg, were observed in sedentary hearts of mice with higher basal levels in female hearts that were altered after physical exercise^38^.

We acknowledge several limitations in our analysis. For example, SHIP study participants are from a rural area in northeast Germany of mostly Caucasian descent. In addition, the analysis of the observational data from the SHIP cohorts cannot determine causality. In addition, albeit we tried to correct for a multitude of potentially confounding factors, residual confounding cannot be ruled out. Nonetheless, a strength of our analysis is the combination of epidemiological and clinical data which suggests the direction of the observed association. We would like to highlight the large sample size available for the cross-sectional analysis between hArg and liver biomarkers.

We summarize that hArg may be a marker of liver dysfunction and should be explored further. Especially the observed sex specific results warrant further investigation.

## Data Availability

SHIP data are publicly available for scientific and quality control purpose. Data usage can be applied for via www.community-medicine.de^18^.
